# Data on Medicare eligibility and cancer screening utilization

**DOI:** 10.1016/j.dib.2016.02.049

**Published:** 2016-02-27

**Authors:** Christian P. Meyer, Christopher B. Allard, Jesse D. Sammon, Julian Hanske, Julia McNabb-Baltar, Joel E. Goldberg, Gally Reznor, Stuart R. Lipsitz, Toni K. Choueiri, Paul L. Nguyen, Joel S. Weissman, Quoc-Dien Trinh

**Affiliations:** a Center for Surgery and Public Health, Brigham and Women’s Hospital, Harvard Medical School, Boston, MA, USA; b Division of Urologic Surgery, Brigham and Women׳s Hospital, Boston, MA, USA; c Department of Medicine, Gastroenterology, Brigham and Women׳s Hospital, Boston, MA, USA; d Department of Surgery, Gastrointestinal and General Surgery, Brigham and Women׳s Hospital, Boston, MA, USA; e Department of Medical Oncology, Dana-Farber Cancer Institute, Brigham and Women׳s Hospital, Harvard Medical School, Boston, MA, USA; f Department of Radiation Oncology, Dana-Farber Cancer Institute, Brigham and Women׳s Hospital, Harvard Medical School, Boston, MA, USA

**Keywords:** Cancer screening, Medicare, Colon cancer, Breast cancer, Prostate cancer, Income strata

## Abstract

Health insurance is associated with increased utilization of cancer screening services. Data on breast, prostate and colorectal cancer screening were abstracted from the 2012 Behavioral Risk Factor and Surveillance System. This data in brief includes two sets of analyses: (i) the use of cancer screening in individuals within the low-income bracket and (ii) determinants for each of the three approaches to colorectal cancer screening (fecal occult blood test, colonoscopy and sigmoidoscopy+fecal occult blood test). Covariates included education attainment, residency, and access to health care provider. The data supplement our original research article on the effect of Medicare eligibility on cancer screening utilization “The impact of Medicare eligibility on cancer screening behaviors” [1].

**Specifications Table**

**Table:** 

Subject area	*Public Health*, *Medicine*
More specific subject area	*Preventive Medicine*, *Cancer Screening*
Type of data	*Tables*
How data was acquired	*Survey*
Data format	*Analyzed*
Experimental factors	*Age as proxy for Medicare eligibility status*
Experimental features	*Cross sectional survey study on the correlation of Medicare eligibility and cancer screening utilization*
Data source location	*USA* (*Nationwide*)
Data accessibility	*Data is within this article*.

**Value of the data**

• Nationally representative data on the utilization of colorectal, breast and prostate cancer screening.
• These data can be compared to other nationally representative surveys.
• These data provide valuable information with regards to cancer screening disparities.

## 1 Data

The Behavioral Risk Factor Surveillance System (BRFSS) is the largest continuously conducted health survey in the US. This joint initiative of the Centers for Disease Control (CDC) and US states/territories is designed to measure behavioral risk factors for the adult population living in households and is administered to a stratified random sample of the U.S. population aged 18 and older. The BRFSS is conducted by landline and cellular telephones in 53 states and territories, providing nationally representative estimates via iterative proportional fitting as a means of weighting. The current methodology minimizes non-response bias and error within estimates. Patients are weighted by age, gender, race/ethnicity, education, marital status, property ownership, and telephone ownership [2]. Cancer screening questions are provided every two years nationally or by discretion of individual state-level questionnaires.

## 2 Experimental design, materials and methods

### 2.1 Study population

Survey data for 2012 were downloaded from the CDC website [2] and extracted. Using the BRFSS codebook [3] we identified survey questions relating to the self-reported use of prostate, colorectal and breast cancer preventive services in the years prior to (ages 60–64) and following (ages 66–70) Medicare eligibility. We excluded persons aged 65 as a washout period. Furthermore, all cases with a previous history of the respective cancers were excluded from analyses to ensure the analysis pertained to screening and not surveillance.

### 2.2 Independent covariates

Covariates included annual household income (<$25,000 and ≥$25,000), health insurance status (Yes vs. No) and access to a regular healthcare provider (HCP) (Yes vs. No). Socio-demographic covariates included age at the time of the survey; education level (Did not graduate High School, Graduated High School, Some College or Technical School and Graduated from College or Technical School); residence location (City Center, Urban/Sub-Urban, and Rural); marital status (Married vs. Never married or Member of Unmarried Couple vs. Divorced, Widowed, Separated).

### 2.3 Statistical methods

Using complex samples methodology, descriptive statistics were calculated for patient demographics. For all point estimates, we calculated 95% confidence intervals (CI׳s) and *p*-values. We used the BRFSS variables _STSTR, _PSU, and _LLCPWT to define strata, cluster, and sample weights respectively. Complex samples multivariable logistic regression was performed to assess for the independent effect of primary predictors on self-reported use of cancer preventive services.

In addition to our original research article [1], in this Data in Brief, we first evaluated demographics within the lowest annual income strata <$25,000 – reflecting the poverty line for four-person households established by the US Census Bureau [4]. Second, the different appropriate approaches for colorectal cancer screening (fecal occult blood test [FOBT], sigmoidoscopy, and colonoscopy) were examined separately. All statistical analyses were performed using the Complex Samples Package for SPSS 20 (IBM, Armonk, NY), with a two-sided significance level set at *p*<0.05. Bonferroni corrections were applied to prevent multiple comparison problems and found our results to be consistent.

## References

[1] Meyer C.P. (2016). The impact of Medicare eligibility on cancer screening behaviors. Prev. Med..

[2] 〈www.cdc.gov/brfss〉

[3] 〈www.cdc.gov/brfss/annual_data/2012/pdf/codebook12_llcp.pdf〉

[4] United States Census Bureau. 〈http://www.census.gov/hhes/www/poverty/data/threshld/〉 (accessed 15.04.15.).

