# Supplementary material for: Data on Medicare eligibility and cancer screening utilization
Source: Data Brief. 2016 Feb 27;7:679–81. doi: 10.1016/j.dib.2016.02.049 (PMC4802418; doi:10.1016/j.dib.2016.02.049)
Supplement: Supplementary file 1 — Supplementary material [file mmc1.docx]

**Data in Brief**

**Table 1.** Baseline characteristics of Non-Medicare and Medicare-eligible population in the low-income group between age 60-70, stratified according to screening service. Note: Cells represent weighted mean (SE) or weighted percentage of sample. All analyses employ survey weights accounting for the Behavioral Risk Factor Surveillance System complex sampling design. Bold numbers indicate p<0.001 and bold numbers with an asterisk * indicate p<0.05 in test of difference by Medicare eligibility.

**Table 2.** Multivariable logistic regression sensitivity analyses of colorectal cancer screening on Medicare eligibility stratified according to the entire cohort and low income population. Bold numbers indicate p<0.001. Asterisk=categories collapsed. HFOB=high focused fecal occult blood test, FOBTS=fecal occult blood test.

|  | Total | | Colorectal cancer screening | | Prostate cancer screening | | Breast cancer screening | |
| --- | --- | --- | --- | --- | --- | --- | --- | --- |
|  | Non-Medicare-eligible | Medicare-eligible | Non-Medicare-eligible | Medicare-eligible | Non-Medicare-eligible | Medicare-eligible | Non-Medicare-eligible | Medicare-eligible |
| Characteristic | (n=12455) | (n=11831) | (n=5199) | (n=6835) | (n=3212) | (n=2315) | (n=5131) | (n=5828) |
| Age (years), M ± SE | 62±0.045 | 68±0.026 | 62 ±0.041 | 68 ±0.31 | 62 ±0.027 | 68 ±0.029 | 62 ±0.038 | 68 ±0.035 |
| Sex (%) |  |  |  |  |  |  |  |  |
| Male | **45.7*** | **41*** | 43.7 | 40.1 | 100 | 100 |  |  |
| Female | **54.3*** | **59*** | 56.3 | 59.9 |  |  | 100 | 100 |
| Education |  |  |  |  |  |  |  |  |
| <High school | **29.6** | **33.8** | **25.3** | **31.6** | **33.4*** | **40.7*** | 27.1 | 30.5 |
| High school | **34.7** | **35.5** | **35** | **35.8** | **34.3*** | **33.5*** | 35.8 | 37.1 |
| Some college | **25.7** | **22.5** | **28.8** | **24.2** | **22.6*** | **17.4*** | 27.7 | 24.7 |
| ≥College | **9.9** | **8** | **10.9** | **8.4** | **9.7*** | **8.5*** | 9.4 | 7.6 |
| Residency |  |  |  |  |  |  |  |  |
| City center | **37.6** | **34.2** | 38.6 | 34.9 | 37.5 | 31.2 | 40.5 | 38.4 |
| Urban | **22.6** | **23.3** | 20.2 | 23.8 | 22.9 | 24 | 21.2 | 21.7 |
| Suburban | **13.6** | **14.7** | 14.2 | 14.1 | 13 | 13.5 | 13.3 | 14.2 |
| Rural | **26.1** | **27.5** | 27 | 27.2 | 26.7 | 31.3 | 25 | 25.9 |
| Access to healthcare provider(%) | **81.9** | **90.4** | **88.8** | **95.1** | **72.6*** | **81*** | **90.9** | **95.8** |
| Insured (%) | **69.5** | **96.4** | **78.4** | **97.5** | **62.7** | **93.9** | **77.4** | **97.9** |

|  | HFOB 1 YR | | Colonoscopy | | FOBTS+Sigmoidoscopy | |
| --- | --- | --- | --- | --- | --- | --- |
|  | Entire cohort | <$25,000 | Entire cohort | <$25,000 | Entire cohort | <$25,000 |
|  | **OR (95% CI)** | **OR (95% CI)** | **OR (95% CI)** | **OR (95% CI)** | **OR (95% CI)** | **OR (95% CI)** |
| Non-Medicare eligibility | ref | ref | ref | ref | ref | ref |
| Medicare eligibility | **1.20 (1.06-1.29)** | **1.22 (1.01-1.47)** | **1.34 (1.25-1.45)** | **1.53 (1.35-1.73)** | 1.08 (0.79-1.48) | 1.56 (0.74-3.30) |
| <High school | ref | ref | ref | ref | ref | ref |
| High school | **1.27 (1.02-1.59)** | **1.47 (1.15-1.78)** | **1.35 (1.16-1.56)** | **1.27 (1.07-1.50)** | 1.81 (0.75-4.37) | 2.43 (0.97-6.09) |
| Some college | **1.34 (1.10-1.70)** | **1.50 (1.14-1.98)** | **1.61 (1.39-1.87)** | **1.58 (1.32-1.90)** | 1.97 (0.80-4.82) | 2.17 (0.78-6.03) |
| ≥College | **1.3 (1.03-1.65)** | **1.50 (1.05-2.14)** | **1.94 (1.67-2.28)** | **1.40 (1.10-1.80)** | **2.95 (1.23-7.08)** | **2.49 (1.01-6.15)** |
| City Center | ref | ref | ref | ref | ref | ref |
| Urban | **0.93 (0.83-1.06)** | 0.89 (0.68-1.16) | 0.96 80.87-1.06) | 0.95 (0.78-1.15) | 0.98 (0.69-1.40) | 0.99 (0.41-2.42) |
| Suburban | **0.79 (0.69-0.91)** | 0.99 (0.70-1.42) | 0.94 (0.84-1.04) | 0.89 (0.73-1.10) | **0.48 (0.32-0.71)** | **0.42 (0.19-0.95)** |
| Rural | **0.78 (0.70-0.87)** | 0.87 (0.68-1.10) | **0.79 (0.73-0.86)** | **0.82 (0.70-0.96)** | **0.45 (0.32-0.63)** | **0.47 (0.23-0.95)** |
| No healthcare provider | ref | ref | ref | ref | ref |  |
| Only one | **2.41 (1.94-3.00)** | **2.25 (1.64-3.08)** | **4.37 (3.83-5.00)** | **3.34 (2.71-4.12)** | *2.11 (0.80-5.53) | - |
| More than one | **2.35 (1.8-3.04)** | **2.26 (1.50-3.40)** | **4.33 (3.63-5.16)** | **3.37 (2.48-4.57)** |  |  |
| <$25,000 | ref |  | ref |  | ref |  |
| >$25,000 | 0.88 (0.77-1-01) |  | **1.63 (1.48-1.79)** |  | 0.95 (0.60-1.51) |  |
